# Inhibitory Effect of Herbal Remedy PERVIVO and Anti-Inflammatory Drug Sulindac on L-1 Sarcoma Tumor Growth and Tumor Angiogenesis in Balb/c Mice

**DOI:** 10.1155/2013/289789

**Published:** 2013-06-27

**Authors:** P. Skopiński, B. J. Bałan, J. Kocik, R. Zdanowski, S. Lewicki, M. Niemcewicz, K. Gawrychowski, E. Skopińska-Różewska, W. Stankiewicz

**Affiliations:** ^1^Department of Histology and Embryology, Center for Biostructure Research, Warsaw Medical University, Chałubińskiego 5, 02-004 Warsaw, Poland; ^2^Department of Immunology, Biochemistry and Nutrition, Warsaw Medical University, Pawińskiego 3a, 01-002 Warsaw, Poland; ^3^Department of Regenerative Medicine, Military Institute of Hygiene and Epidemiology, Kozielska 4, 01-163 Warsaw, Poland; ^4^Biological Threats Identification and Countermeasure Center of the Military Institute of Hygiene and Epidemiology, Lubelska 2, 24-100 Pulawy, Poland; ^5^Department of Gynecological Oncology and Oncology, Medicover Hospital, Aleja Rzeczypospolitej 5, 02-972 Warsaw, Poland; ^6^Pathology Department, Center for Biostructure Research, Warsaw Medical University, Chałubińskiego 5, 02-004 Warsaw, Poland; ^7^Department of Microwave Safety, Military Institute of Hygiene and Epidemiology, Kozielska 4, 01-163 Warsaw, Poland

## Abstract

Anticancer activity of many herbs was observed for hundreds of years. They act as modifiers of biologic response, and their effectiveness may be increased by combining multiple herbal extracts . PERVIVO, traditional digestive herbal remedy, contains some of them, and we previously described its antiangiogenic activity. Numerous studies documented anticancer effects of nonsteroidal anti-inflammatory drugs. We were the first to show that sulindac and its metabolites inhibit angiogenesis. In the present paper the combined *in vivo* effect of multicomponent herbal remedy PERVIVO and nonsteroidal anti-inflammatory drug sulindac on tumor growth, tumor angiogenesis, and tumor volume in Balb/c mice was studied. These effects were checked after grafting cells collected from syngeneic sarcoma L-1 tumors into mice skin. The strongest inhibitory effect was observed in experimental groups treated with PERVIVO and sulindac together. The results of our investigation showed that combined effect of examined drugs may be the best way to get the strongest antiangiogenic and antitumor effect.

## 1. Introduction

 Tumor angiogenesis, the development of new blood vessels within the primary tumor or in metastasis hotspots, is an essential process for the growth and progression of metastases. Both innate and adaptive immune systems are involved in positive and negative regulations of this process. All over the world various research teams are conducting studies to find agents, which could potentially be able to inhibit the process. Despite the discovery of many such agents, the need for further research exists, because the angiogenesis inhibitors obtained so far are either very costly to synthesize, or they exhibit a number of side effects. Angiogenesis is a complex process with many contributing factors and one regulated by endogenic stimulators and inhibitors. Among the scientists involved in such studies a common conviction exists; that is the single angioinhibitors are not able to suppress the process effectively. Most antiangiogenic natural health products block new vessel formation at multiple molecular levels. Thus the need to recourse to low-toxic vegetal compounds and studies of combined effects of a few low-dosage compounds with different modes of action on the processes of angiogenesis in tumor growth. Many phytochemicals and diet derivatives are able to exert chemopreventive and antitumor activity targeting the tumor environment and inflammatory angiogenesis [1–3].

 PERVIVO is a digestive herbal remedy, mixture of 27 herbs alcoholic extracts dissolved in 32% ethyl alcohol, some of them traditionally used as anti-inflammatory agents also possessing antimicrobial or anti-tumor properties. We were the first to show angioinhibitory effect of this remedy in local cutaneous model of tumor angiogenesis [4]. The most important anti-tumor substance in PERVIVO is *Radix zingiberis* (ginger). Ginger has a long history of medicinal use dating back 2500 years. The anticancer properties of ginger are connected mainly with gingerols, shogaols, and zingerone [5]. 6-gingerol, a natural component of ginger, was shown to inhibit growth of colon cancer cells via induction of G2/M arrest [6]. The second ginger component, 6-shogaol, induced apoptosis in human hepatocellular carcinoma cells and exhibited anti-tumor activity *in vivo* through endoplasmic reticulum stress [7]. Ginger treatment suppressed the proliferation and colony formation in breast cancer cell lines [8]. Chakraborty et al. observed the *in vitro* effect of 6-gingerol on HeLa cells. Their results suggest that 6-gingerol has potential to bind with DNA and induce cell death by autophagy and caspase-3-mediated apoptosis [9]. Silva et al. demonstrated specific antiproliferative activities of gingerols against MDA-MB-231 tumor cell line [5]. Shogaols are dehydration products of corresponding gingerols during storage or thermal processing. Shogaols have stronger inhibitory effect than gingerols on growth of cancer cells, arachidonic acid release, and nitric oxide (NO) synthesis [10]. In experiments of Weng et al. both 6-shogaol and 6-gingerol effectively inhibited invasion and metastasis of hepatocellular carcinoma but through diverse molecular mechanisms. Both of them regulate MMP-2/-9 transcription. 6-Gingerol directly decreased expression of urokinase plasminogen activator and 6-shogaol indirectly by upregulation plasminogen activator inhibitor [11] and via blockade of nuclear factor-*κ*B activation [12]. 

It was also documented that ginger and its compounds inhibit angiogenesis *in vitro* and *in vivo* [13–15]. 

The next PERVIVO ingredient, *Artemisia absinthium,* inhibited TNF alpha production and accelerated healing of patients with Crohn's disease [16]. Artemisinin, active antimalarial compound isolated from herbs belonging to *Artemisia* species, dihydroartemisinin (DHA), a semisynthetic derivative of artemisinin, and hispidulin, small flavonoid from *Artemisia vestita*, inhibited growth of pancreatic, prostate, and ovarian cancer cells and were shown to be cytotoxic to cancer cells through induction of apoptosis. The antiangiogenic effect of artemisinin *in vitro* and *in vivo* was also described [17–25]. 

The claims concerning anti-tumor activity of some other PERVIVO compounds are not largely supported by scientific evidence as yet. Radix *Angelicae sinensis *is a medicinal herb and health food supplement that has been widely used in Asia for centuries. Cytotoxicity against tumor cell lines of its extracts, epigenetic modifications of cancer oncogenes, and tumor suppressor genes were described. It was also reported that Radix *Angelicae sinensis* may be a potential source of glutathione S-transferase inhibitors and counteract multidrug resistance [26–30]. One report described antiproliferative activity of *Gentiana triflora* root extract on cultured and implanted tumor cells [29]. Fruit oils of *Litsea cubeba* from Taiwan exhibited cytotoxic activity against human lung, liver, and oral cancer cells *in vitro* [30, 31]. Galangin, flavonol present in *Alpinia galanga* rhizome, induced apoptosis of cancer cells [32, 33]. Extract of Radix Liquiritiae (licoricidin from *Glycyrrhiza uralensis*) inhibited the metastatic potential of human prostate cancer cells [34].

Most components of PERVIVO possess antimicrobial activity. Essential oil and decoction of Carlinae Radix (*Carlina acanthifolia* L.) showed significant antimicrobial effect against *Staphylococcus aureus *[35]. Compounds from *Artemisia *are antiplasmodial and antitrypanosomal drugs, and could be an alternative drug against trichinellosis [36–38]. Compounds from *Alpinia galanga* are potent inhibitors for the influenza virus replication [39] and exhibit significant activity *in vitro* against promastigotes of *Leishmania donovani* [40]. Phenylpropanoids of *Alpinia galanga* was efflux pump inhibitors in *Mycobacterium smegmatis* mc^2^ 155 cells [41]. Essential oils from *Litsea cubeba* contain fungicidal and antibacterial terpenoids [42, 43]. Sesquiterpene lactones from *Inula helenium* root essential oil exhibited antistaphylococcal activity [44].

Epidemiological studies have suggested that the use of nonsteroidal anti-inflammatory drugs (NSAIDs) may reduce the risk of cancer. Numerous studies have been devoted to the action of such anti-inflammatory agents as aspirin, indomethacin, piroxicam, and sulindac. The anticancer effect of NSAIDs was mainly found to result from their proapoptotic and antiproliferative effects which, therefore, restrict tumorigenesis [45–49].

We were the first to show that sulindac and its metabolites (sulindac sulfone and sulindac sulphide), described previously by other authors as pro-apoptotic anticancer drugs [50, 51], inhibit tumor growth and angiogenesis induced in mice skin by cells isolated from murine L-1 sarcoma as well as angiogenesis induced by cells collected from human kidney and pulmonary cancers [52–55]. The aim of the present work was to study the combined *in vivo* effect of multicomponent herbal remedy PERVIVO and non-steroidal anti-inflammatory drug sulindac on tumor growth, tumor angiogenesis, and tumor volume in Balb/c mice. These effects were checked after grafting of cells collected from syngeneic sarcoma L-1 tumors into mice skin. 

## 2. Material and Methods

### 2.1. Drugs

 PERVIVO (Richard Bittner GmbH, Weitensfeld, Austria) is herbal remedy composed of 27 herbal extracts dissolved in 32% ethyl alcohol (Table 1). Sulindac (Sudaklin, Polpharma SA, Starogard Gdański, Poland) is nonsteroidal anti-inflammatory drug.

### 2.2. Mice

The study was performed on female, 8–10-week old inbred Balb/c mice, about 20 g of body mass, delivered from the Polish Academy of Sciences Breeding Colony. For all performed experiments animals were handled according to the Polish law on the protection of animals and NIH (National Institutes of Health) standards. All experiments were accepted and conducted according to ethical guidance of Local Bioethical Committee. Mice were housed 4-5 per cage and maintained under conventional conditions (room temperature 22.5–23.0°C, relative humidity 50–70%, and 12 h day/night cycle) with free access to standard rodent diet and water. 

### 2.3. Sarcoma L-1 Tumor Cells

L-1 sarcoma cells from *in vitro* culture stock were delivered from Warsaw's Oncology Center collection, passaged *in vivo,* and grafted subcutaneously (for evaluation of tumor growth) or intradermally (for evaluation of angiogenic activity) to syngeneic Balb/c mice. 

### 2.4. Treatment of Mice with PERVIVO and/or Sulindac

Mice received orally by Eppendorff pipette 20 *μ*L of PERVIVO with 20 *μ*L of water, or sulindac 0.6 mg, both drugs, for 3 days in cutaneous tumor-induced angiogenesis (TIA) test or for 14 days in evaluation of the effect of drugs on tumor growth. These doses correspond to 10 mL of PERVIVO and 300 mg of sulindac given to 70 kg person (applying the counter 7 for differences between mouse and human in relation of the surface to body mass). Control mice were fed with 40 *μ*L of 16% ethyl alcohol.

### 2.5. Preparation of Tumor Cells after *In Vivo* Passage

Briefly, sarcoma L-1 cells from *in vitro* stock were grafted (10^6^/0.1 mL) subcutaneously into subscapular region of Balb/c mice. After 14 days, the tumors were excised, cut to smaller pieces, rubbed through sieve, and suspended in 5 mL of PBS. The suspension was left for 10 min at room temperature. 

After sedimentation, the supernatant was collected and centrifuged for 10 min at 1500 rpm. Obtained sarcoma cells were washed once with PBS for 10 min, then centrifuged at 1500 rpm, and resuspended in Parker medium in concentration of 4 × 10^6^ cells/mL (for tumor-induced angiogenesis) or 10^7^ cells/mL (for experiments with tumor growth). Viability of cells was about 95% of living cells as estimated by trypan-blue method.

### 2.6. Cutaneous Angiogenesis Assay (Tumor-Induced Angiogenesis (TIA) Test)

**Figure 1:**
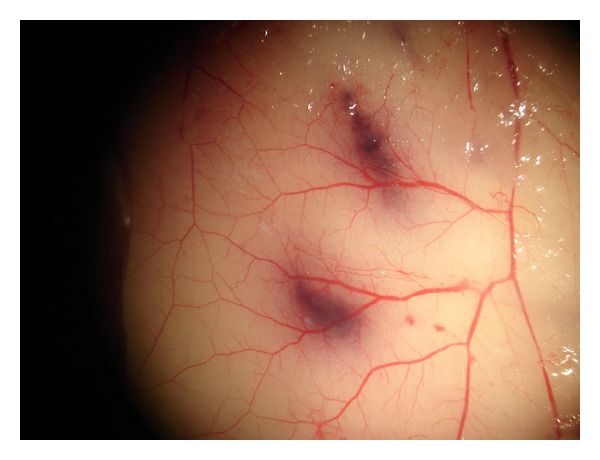
Typical picture of newly-formed blood vessels.

Multiple 0.05 mL samples of 200 thousands of cells were injected intradermally into partly shaved, narcotised Balb/c mice (at least 3-4 mice per group). In order to facilitate the localization of cell injection sites later on, the suspension was colored with 0.1% of trypan blue. Mice were fed with drugs for 3 days. After 72 hours, mice were sacrificed with lethal dose of Morbital (Biowet, Puławy). All newly formed blood vessels were identified and counted in dissection microscope, on the inner skin surface, at magnification of 6x, in 1/3 central area of microscopic field. Identification was based on the fact that the new blood vessels are thin, directed to the point of cells injection and (or) differ from the background vasculature in their tortuosity and divarications (Figure 1).

All experiments were performed in anaesthesia (3.6% chloral hydrate, Sigma-Aldrich), 0.1 mL per 10 g of body mass.

### 2.7. Subcutaneous Tumor Growth Assay

Suspensions of sarcoma cells were grafted (2 millions of cells) subcutaneously into mice. On the day of cells grafting and on the following 13 days mice were fed PERVIVO, sulindac, PERVIVO and sulindac, or diluted ethyl alcohol as a control. Mice were observed during these days, with number of appearing tumors noted at 7, 9, and 11 days after grafting. At days 9 and 14 the tumors volume was measured with electronic caliper (The Fowler Ultra-Cal Mark III caliper). After 14 days mice were sacrificed. 

### 2.8. Morphological Examination

Morphological examination was done using light-microscopic analysis. Immediately after resection, tumor specimens were fixed in 10% formaldehyde solution. After fixation the specimens were dehydrated in increased concentration of alcohol and embedded in paraffin. Paraffin tissue block was sectioned on 4 *μ*m thin sections. The specimens were stained by hematoxyline and eosine.

#### 2.8.1. Statistical Evaluation of the Results

Evaluation of the results was performed by chi-square test and one-way ANOVA with Bartlett's test for equal variances, and the significance of differences between the groups was verified with a Bonferroni Multiple Posttest (GraphPad Prism).

## 3. Results

**Figure 2:**
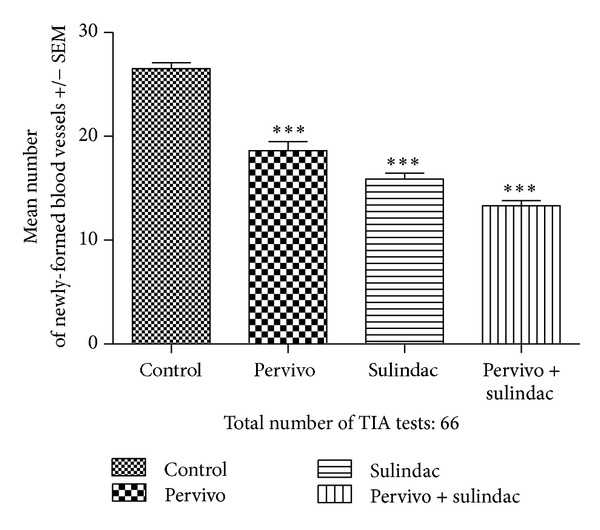
Inhibitory effect of PERVIVO and sulindac on neovascular reaction induced in mice skin after grafting L-1 sarcoma cells. ***P < 0.001.

**Figure 3:**
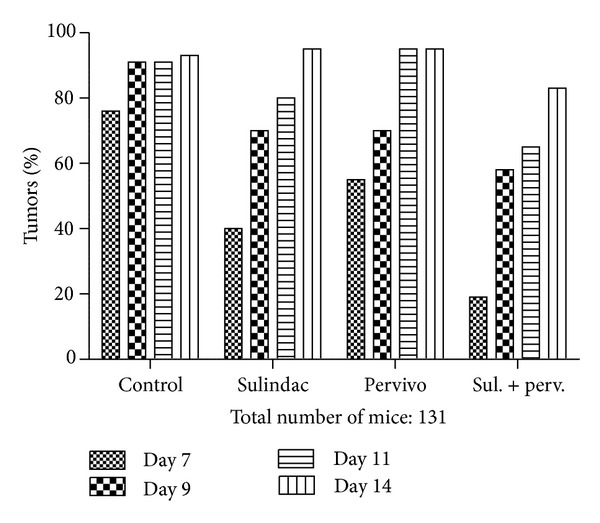
% of mice with measurable tumors in various days after L-1 sarcoma cells grafting.

Histological examination revealed no major differences between tumors collected from control and experimental groups of mice. The dominant picture was mass of poorly differentiated atypical cells with features of sarcoma. As shown in Figure 2 and Table 2 the number of newly formed vessels that were induced by the tumor presence was decreased both by PERVIVO and sulindac, with the effect of sulindac comparatively stronger. Joint application of both drugs resulted in even stronger effect than treatment with separate compounds. The influence of the study drugs on measurable tumor emergence in respective days after sarcoma load inoculation is shown in Figure 3 and Table 3. Similar to the antiangiogenic effect, the influence of sulindac alone was greater than this by PERVIVO, and the common appliance of both drugs resulted in even greater delay in measurable tumor appearance. The time of the strongest impediment of tumor growth was similar in case of all study settings (day 7). The effect was intermittent, and only after application of both drugs together, the measurable tumors have not developed in all animals.

**Figure 4:**
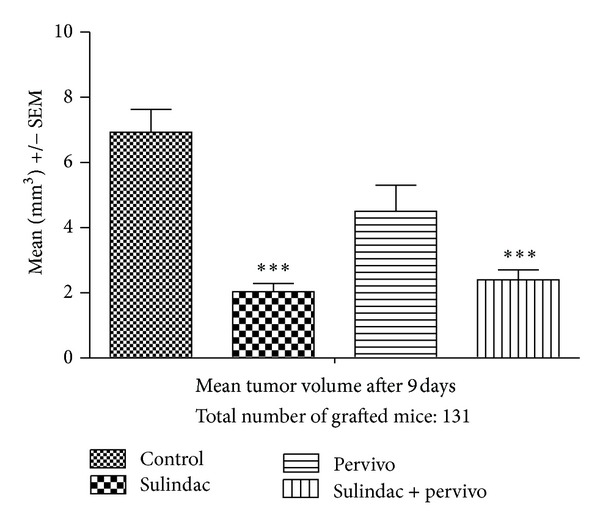
Mean tumor volume 9 days after Sarcoma L-1 cells grafting.

**Figure 5:**
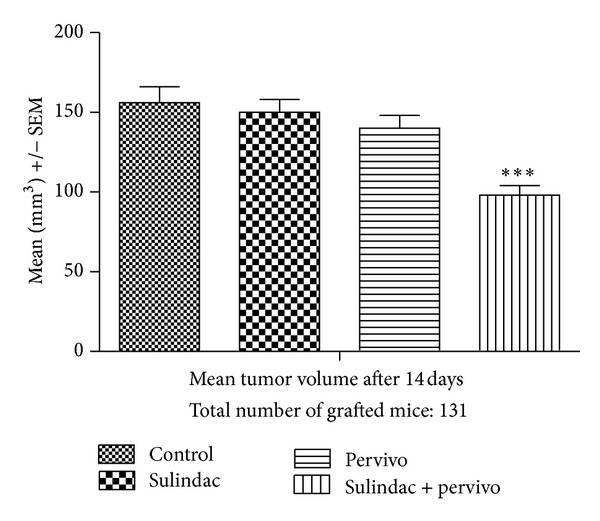
Mean tumor volume 14 days after L-1 sarcoma cells grafting ***P < 0.001.

The influence of the study drugs on the mean tumor volume at the respective study time points is shown in Figures 4 and 5. The mean volume was statistically significantly lower at day 9 after administration of sulindac and, to the lesser extent, by both drugs (Table 4). The effect was not clearly defined in the case of PERVIVO. The mean tumor volume was lower than in placebo group but the difference was not significant. At day 14 only in the group that received both drugs the mean tumor volume was significantly lower compared to the placebo (Figure 5 and Table 5).

## 4. Discussion

L-1 sarcoma tumor arose spontaneously in the lung of Balb/c mouse and was described by Przemysław Janik from Warsaw Oncology Center (58). This tumor has been maintained since then by subcutaneous serial passages in Balb/c mice and frozen and stored in Warsaw Oncology Center Tissue Collection. Isolated L-1 cells from tumors were adapted to grow *in vitro*. 

 L-1 sarcoma cells from culture, after grafting to animals, form tumors in *in vivo* conditions.

L-1 sarcoma is a perfect experimental model for assessing the impact of various substances upon the tumor growth and activity of its cells. 

Previously, we used L-1 sarcoma cells for evaluation of pro- and antiangiogenic activity of various substances of synthetic and natural origin. We were the first to report the anti-angiogenic activity of sulindac and its metabolites, as well as that of theobromine, catechins in the cacao tree seeds, salidroside and rosavin isolated from the *Rhodiola rosea* and the *Rhodiola quadrifida*, convallamaroside isolated from the *Convallaria majalis* rhizome, alkylglycerols found in the shark liver oil, and other substances of natural origin [56–62].

We also demonstrated inhibitory effect of these substances in the angiogenic reaction induced in the mouse skin by cells or tissue homogenates obtained from surgically removed human cancers of the lung, kidney, ovary, and urinary bladder. We also proved the synergic anti-angiogenic activity of particular combinations of synthetic and natural inhibitors in relation to the reaction induced in mice skin with the human serum. We demonstrated the synergic activity of small doses of sulindac, convallamaroside, ursolic acid and epigallocatechin gallate (EGCG) in inhibition of cutaneous angiogenesis induced in mice by intracutaneous administration of serum obtained from patients with diabetic retinopathy. Additionally, we demonstrated that human recombined cytokines (VEGF, bFGF, IL-8, and IL-18) induced the cutaneous angiogenesis inhibited by natural and synthetic inhibitors. Sulindac and its metabolites inhibited angiogenesis induced by bFGF and IL-18 and did not affect the angiogenic VEGF and IL-8 activity [63, 64].

Our experience, dealing with the question of angiogenic inhibitors, indicates that it is crucial to use simultaneously multiple anti-angiogenic factors of different handle points in the treatment. Many inhibitors display a synergic activity; therefore, their appropriate combinations may significantly reduce drugs doses and their possible side effects [65]. One of the examples of remarkable synergistic effect is inhibition of both the neovascularization and growth of tumor in head and neck squamous cell carcinoma by retinoic acid and interferon alfa by different mechanisms of action. Tumor cells treated by interferon alfa stopped secretion of interleukin 8, the major angiogenic factor, while retinoic acid caused them to secrete an inhibitor of angiogenesis [66]. There is also a proof for synergism between cyclooxygenase inhibitors and cytotoxic chemotherapy drugs in inhibition of angiogenesis. Both celecoxib and 5-fluorouracil impaired angiogenesis by inhibiting vascular endothelial growth factor (VEGF). Additionally celecoxib influenced interferon gamma that has a pivotal role in tumor suppression [67]. Thalidomide has shown synergistic effect with low, nontoxic dose of cisplatin. It was shown that this effect is related to antiangiogenic influence of thalidomide on different tumor-related mediators: VEGF, basic fibroblast growth factor, hepatocyte growth factor, and IL-8 [68]. All evidence, of which examples are noted above, may lead to the concept of integrative approach of managing a patient with cancer. Cytotoxic drugs that are used as “golden standard” in cancer chemotherapy currently have high toxicity in therapeutic doses. The research is ongoing on combining them with compounds that target multiple biochemical pathways in processes that promote different aspects of cancer development, angiogenesis being one of the most important. These are i.a. natural health products that may be used as biological response modifiers and adaptogens, providing quality assurance of extracts is assured, and the effectiveness of combinations is proved in clinical trials.

We described inhibitory *in vivo* effect of two combinations of natural substances on angiogenesis and L-1 sarcoma growth in Balb/c mice. First of them was a composition of two Scandinavian folk medicine products, Greenland shark liver oil (rich in alkoxyglycerols) and arctic birch ashes, supplemented with squalene. All these substances alone and in combination significantly diminished cutaneous angiogenesis induced by tumor cells and tumor growth [69]. The second remedy composed of *Echinacea purpurea* extract, *Allium sativum* extract, and cocoa [70]. Again, a significant inhibitory effect on tumor angiogenesis and L-1 sarcoma growth was observed. 

Other authors reported enhancing effect of theanine, a component of green tea leaves, on the antitumor activity of adriamycin [71], inhibition of liver metastasis of human pancreatic carcinoma by angiogenesis inhibitor TNP-470 (analog of fumagillin derived from *Aspergillus fumigatus*) in combination with cisplatin in nude mice [72], and augmented antitumor effects of combination therapy cisplatin/TNP-470/IL-12 in melanoma (B6D2F1 mice) and colon carcinoma (Balb/c mice) [73]. Recently, anticancer activity of noscapine, an opioid alkaloid in combination with cisplatin in human nonsmall cell lung cancer, *in vitro* and *in vivo*, in murine xenograft model was reported [74].

Selvendiran et al. [75] reported the results of *in vitro* and *in vivo* experiments with HO-3867, a curcumin analog, combined with cisplatin. This compound sensitized cisplatin-resistant ovarian carcinoma, leading to therapeutic synergy through STAT3 inhibition. Similar effect was obtained for dihydroartemisinin (derivative of *Artemisia* compound artemisinin, one of the PERVIVO components) which induced apoptosis and sensitized human ovarian cancer cells to carboplatin therapy [76] and improved the efficiency of chemotherapeutics (cisplatin, cyclophosphamide) in lung carcinomas *in vivo* and *in vitro* [77]. Lu et al. reported synergistic action of the C-Jun N-terminal Kinase (JNK) inhibitor and dihydroartemisinin in apoptosis induction through accelerating Bax translocation into mitochondria in human lung adenocarcinoma cells [78].

Suganuma et al. described synergistic proapoptotic effects of epigallocatechin gallate and epicatechin on human lung cancer cell line PC-9 *in vitro*. This effect was increased by sulindac or tamoxifen [79]. Recently, beneficial effect of sulindac, in combination with dimethylamino parthenolide (nuclear factor-*κ*B inhibitor) and gemcitabine, in genetically engineered mouse model of pancreatic cancer was described [80]. It was revealed that sulindac sulfide can inhibit tumor cell invasion *in vitro* at concentrations less than those required to inhibit tumor cells growth, by suppressing NF-*κ*B-mediated transcription of microRNAs [81]. Novel sulindac derivatives that do not inhibit COX-1 and COX 2 suppressed colon tumor cell growth *in vitro* by inhibiting cGMP phosphodiesterase and *β*-catenin transcriptional activity [82] and inhibited *in vivo* malignant pleural adenocarcinoma dissemination in mice [83].

## Figures and Tables

**Table:** (a) Active components

Radix *Angelicae *	1.360 g
Radix *Gentianae *	0.500 g
*Menyanthis folium *	0.120 g
Herba Absinthii	0.035 g
Radix *Zingiberis *	0.015 g
Camphora racemica	0.950 g
Theriak	0.970 g

**Table:** (b) Additional

Fructus Anisi stellati	0.046 g
Myrrha	0.700 g
Herba Cardui benedicti	0.015 g
Herba Centaurii	0.013 g
Flos Caryophylli	0.030 g
Radix Galangae	0.014 g
Radix Liquiritiae	0.170 g
Radix Calami	0.047 g
Radix Helenii	0.020 g
Radix Zedoariae	1.380 g
Manna	1.360 g
Flos Verbasci	0.014 g
Radix Carlinae	0.680 g
Semen Myristicae	0.280 g
Herba Ivae moschatae	0.006 g
Radix Iridis	0.005 g
Pericarpium Aurantii amari	0.031 g
Cortex Curacao	0.038 g
Fructus Cubebae	0.017 g
Cortex Aurantii dulcis	0.011 g

**Table:** (a)

One-way analysis of variance	
*P* value*	<0.0001
*P* value summary	***
Are means signif. different? (*P* < 0.05)	Yes
Number of groups	4
*F*	88.90
*R* square	0.8114

**Table:** (b)

Bonferroni test	Mean diff.	*t*	Significant? *P* < 0.05?	Summary
Control versus PERVIVO	7.900	8.926	Yes	***
Control versus sulindac	10.60	11.98	Yes	***
Control versus PERVIVO + sulindac	13.20	15.19	Yes	***
PERVIVO versus sulindac	2.700	2.854	Yes	*
PERVIVO versus PERVIVO + sulindac	5.300	5.691	Yes	***

*P < 0.05, ***P < 0.001.

**Table 3:** Statistical analysis of the results presented inFigure 3.

Chi-square	
Chi-square, df.	23.55, 9
*P* value	0.0051
*P* value summary	∗∗
One- or two-sided	NA
Statistically significant? (alpha < 0.05)	Yes
Data analyzed	
Number of rows	4
Number of columns	4

**P < 0.01.

**Table:** (a)

One-way analysis of variance	
*P* value	<0.0001
*P* value summary	∗∗∗∗
Are means. signif. different? (*P* < 0.05)	Yes
Number of groups	4
*F*	14.42
*R* square	0.3223

**Table:** (b)

Bonferroni test	Mean diff.	*t*	Significant?	*P* < 0.05?
Control versus sulindac	4.890	6.973	Yes	∗∗∗
Control versus PERVIVO	2.430	3.465	No	ns.
Control versus sulindac + PERVIVO	4.530	8.125	Yes	∗∗∗
Sulindac versus PERVIVO	−2.460	2.892	No	ns.
Sulindac versus sulindac + PERVIVO	−0.3600	0.4886	No	ns.
PERVIVO versus sulindac + PERVIVO	2.100	2.850	No	ns.
Sulindac versus sulindac + PERVIVO	−0.3600	0.4886	No	ns.
PERVIVO versus sulindac + PERVIVO	2.100	2.850	No	ns.

***P < 0.001, ****P < 0.0001.

**Table:** (a)

One-way ANOVA				
Source of variation	% of total variation	*P* value	*P* value summary	Significant?
Interaction	1.53	0.0101	∗	Yes
Drug	4.25	<0.0001	∗∗∗	Yes

**Table:** (b)

Bonferroni test	Mean difference	*t*	*P* value	Summary
Control versus sulindac	−6.000	0.5758	*P* > 0.05	ns
Control versus PERVIVO	−16.00	1.535	*P* > 0.05	ns
Control versus sulindac + PERVIVO	−58.00	6.935	*P* < 0.001	∗∗∗
Sulindac versus sulindac + PERVIVO	−52.00	4.990	*P* < 0.001	∗∗∗
PERVIVO versus sulindac + PERVIVO	−42.00	4.030	*P* < 0.001	∗∗∗
Control versus sulindac	−6.000	0.5758	*P* > 0.05	ns
Control versus PERVIVO	−16.00	1.535	*P* > 0.05	ns
Control versus sulindac + PERVIVO	−58.00	6.935	*P* < 0.001	∗∗∗
Sulindac versus sulindac + PERVIVO	−52.00	4.990	*P* < 0.001	∗∗∗
PERVIVO versus sulindac + PERVIVO	−42.00	4.030	*P* < 0.001	∗∗∗

*P < 0.05, ***P < 0.001.
